# Expanded non-invasive prenatal testing offers better detection of fetal copy number variations but not chromosomal aneuploidies

**DOI:** 10.1371/journal.pone.0312184

**Published:** 2025-01-24

**Authors:** Shaozhe Yang, Yuan Zhuang, Junfeng Li, Xiuhong Fu

**Affiliations:** 1 Henan Key Laboratory of Fertility Protection and Aristogenesis, Luohe Central Hospital, Luohe, Henan Province, People’s Republic of China; 2 Warren Alpert Medical School, Brown University, Providence, RI, United States of America; Universita degli Studi di Roma Tor Vergata, ITALY

## Abstract

**Purpose:**

To evaluate the clinical performance of expanded non-invasive prenatal testing (NIPT-plus) and compare its effectiveness in screening for chromosomal aneuploidies with that of NIPT.

**Methods:**

Screening results, confirmatory invasive testing results, and follow-up data from pregnant women who underwent either NIPT (6792 cases) or NIPT-Plus (5237 cases) testing at Luohe Central Hospital, China, from January 2019 to June 2023 were collected. The positive predictive value (PPV), sensitivity, specificity, and other indicators for different types of chromosomal abnormalities in NIPT/NIPT-plus screening were calculated. The willingness of pregnant women with various types of abnormalities to undergo confirmatory invasive testing and the proportion of pregnancy terminations were investigated.

**Results:**

The average number of unique reads in NIPT-plus samples was 5.26 times greater than that in NIPT samples. There was no significant difference in the PPV or positive rate between NIPT-plus and NIPT for screening chromosomal aneuploidies. Compared with the low-risk group, the high-risk group had a greater PPV; however, in the NIPT-plus group, there was no significant disparity in the PPV between the low-risk and high-risk groups. Compared with rare autosomal aneuploidies (RAAs), pregnant women had a higher rate of confirmatory invasive testing for common trisomies, sex chromosomal abnormalities (SCAs), and copy number variations (CNVs). However, the rate of pregnancy termination for common trisomies, RAAs, and CNVs was higher than that for SCAs.

**Conclusion:**

By enhancing sequencing data, NIPT-plus can effectively screen for CNVs as well as chromosomal aneuploidies. However, NIPT-plus does not have an advantage over standard NIPT in screening for chromosomal aneuploidies.

## Introduction

Genetic defects are the leading factor behind miscarriage, stillbirth, severe congenital abnormalities and severe disabilities [1]. In China, the rate of birth defects is estimated to be approximately 5.6% [2]. Chromosomal abnormalities, such as common trisomies, including trisomy 21 (T21), trisomy 18 (T18), and trisomy 13 (T13); sex chromosome aneuploidies (SCAs); and chromosomal copy number variations (CNVs), are the main causes of birth defects [3, 4]. The development of copy number variation sequencing (CNV-seq) and chromosomal microarray analysis (CMA) in recent years has led to increased attention to the detection and prevention of pathogenic CNVs [5]. Some CNVs can result in severe microdeletion and microduplication syndromes (MMSs) [6]. MMSs have a high occurrence rate, and this rate is not related to maternal age [7]. Reports suggest that the occurrence of fetal MMSs in pregnant women with normal chromosomes can reach 1–1.7%. This includes conditions such as DiGeorge syndrome, Williams syndrome, and Prader–Willi syndrome, which can cause intellectual disabilities and developmental delays [8].

Prenatal screening and diagnosis are the main measures used to prevent birth defects. Traditional prenatal screening, which is based on maternal serum biomarkers, can screen for T21, T18, and open neural tube defects at a lower cost. However, it is gradually being phased out because of its lower detection rate and higher false-positive rate (FPR) [9]. While fetal chromosome karyotyping analysis and/or CNV detection following amniocentesis or chorionic villus sampling is the gold standard for diagnosing fetal chromosomal abnormalities, the procedure poses a risk of miscarriage or fetal harm, making it unpopular among the majority of pregnant women [10].

Non-invasive prenatal testing (NIPT) utilizing next-generation sequencing (NGS) for cell-free fetal DNA (cffDNA) and bioinformatics analysis has rapidly gained popularity since its first application in 2011 [11] because of its non-invasive, simple, and accurate advantages [12]. NIPT has demonstrated high positive predictive value (PPV), sensitivity, and specificity and an extremely low false-negative rate (FNR) in screening for chromosomal aneuploidies [13–17]. Currently, all NIPT platforms use basic read counting for each chromosome and simple statistical methods, such as the Z test, to identify aneuploidies [18]. Essentially, with increasing sequencing depth, this method has the ability to detect fetal chromosomal CNVs [19]. As a result, expanded noninvasive prenatal testing (NIPT-plus) has been created, allowing for the simultaneous screening of chromosomal aneuploidies and CNVs [20–22].

Despite some studies indicating the value of NIPT-plus in testing for CNVs and other diseases [20–23], there is still much debate among doctors regarding the performance of NIPT-plus. (1) NIPT-plus shows notable variance in accuracy when identifying rare autosomal aneuploidies (RAAs) and CNVs [24, 25]. (2) The PPVs for RAAs and CNVs in NIPT-plus are lower than those for common trisomies, and false-positive (FP) results may lead to incorrect termination of pregnancy (TOP). (3) Due to the absence of uniform quality control standards, NIPT-plus may yield different detection outcomes depending on the testing platform and bioinformatics algorithm used. (4) Many obstetricians wonder if NIPT-plus can enhance the detection efficacy of common trisomies and SCAs, even with the added sequencing data output and testing costs.

In this research, both NIPT (6792 cases) and NIPT-plus (5237 cases) were reviewed and analyzed for their clinical data, screening efficiency, pregnant women’s willingness for confirmatory invasive testing, results of confirmatory invasive testing, and pregnancy outcomes. The main testing indicators for NIPT and NIPT-plus were compared. We also analyzed the willingness of pregnant women with various types of chromosomal abnormalities to undergo confirmatory invasive testing and the TOP percentage. The findings of this study are beneficial for the widespread use of NIPT-plus and will assist obstetricians and pregnant women in choosing between NIPT and NIPT-plus.

## Materials and methods

### Subjects

**Fig 1:**
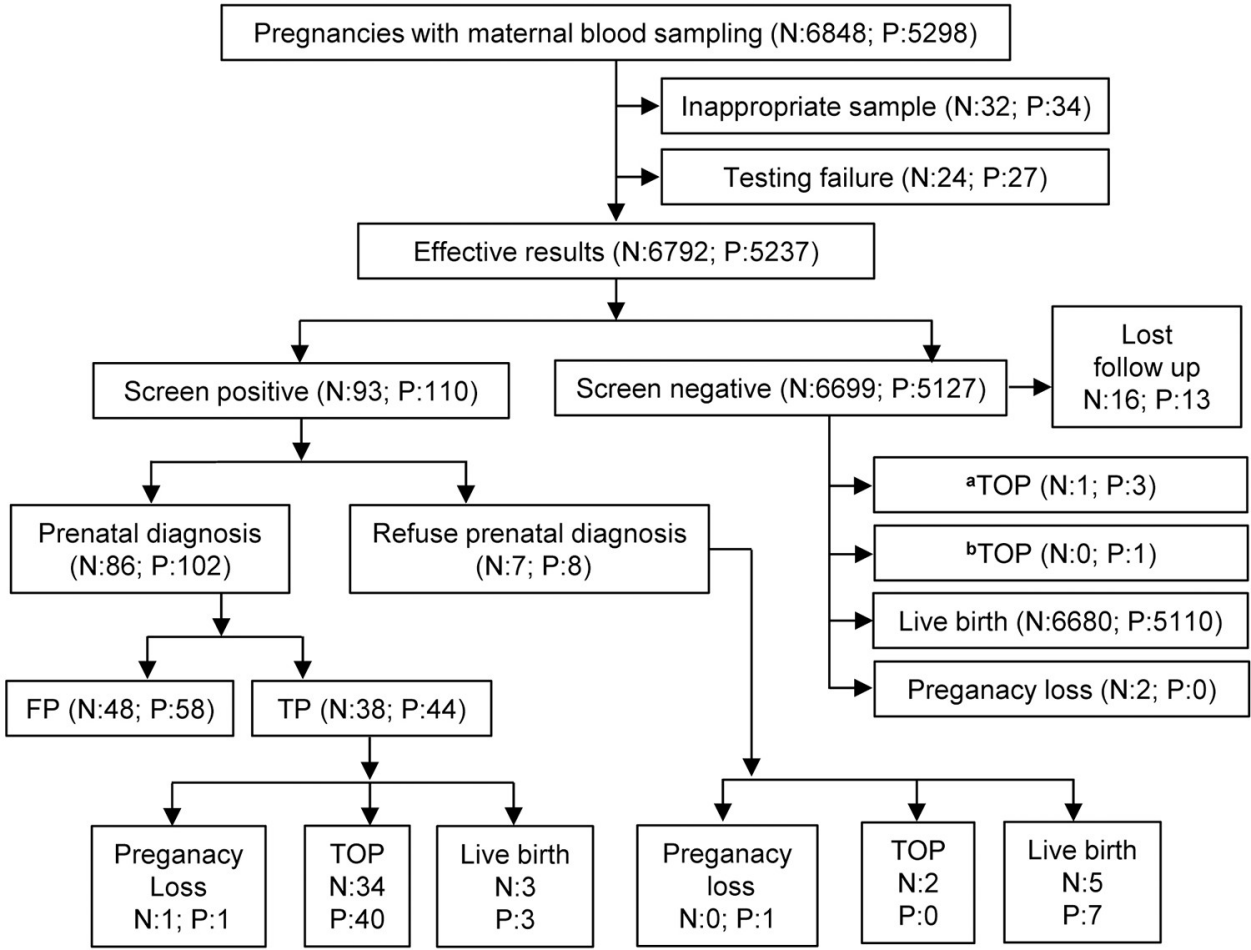
Flowchart of the NIPT/NIPT-plus results and outcomes of pregnant women. N, NIPT, non-invasive prenatal testing; P, NIPT-plus, expanded non-invasive prenatal testing; FP, falsepositive; TP, true positive; TOP, termination of pregnancy; ^a^TOP, TOP due to abnormal ultrasound; ^b^TOP, TOP of a healthy fetus.

This retrospective study included 6848 pregnant women who received NIPT and 5298 pregnant women who received NIPT-plus testing at Luohe Central Hospital in China from January 2019 to June 2023. The data exclusion criteria included the following: (1) incomplete clinical information (NIPT: 32; NIPT-plus: 34) and (2) two consecutive test failures due to a low fetal DNA fraction or poor quality of blood (NIPT: 24; NIPT-plus: 27), resulting in a total of 6792 cases of NIPT and 5237 cases of NIPT-plus included in this study (Fig 1). Pregnant women were registered, and their blood was drawn from Luohe Central Hospital (a prenatal diagnosis center) and seven collaborating prenatal screening institutions. Clinical data of the research subjects, including names, ages, gestational weeks, ethnicity, obstetric history, mode of delivery, fetal ultrasound examination results, NIPT/NIPT-plus results, confirmatory invasive testing results, pregnancy outcomes, and newborn physical examination reports, were collected.

All pregnant women were provided genetic counseling, were made aware of the potential for both FP and false-negative (FN) results, and then proceeded to sign written informed consent forms. This research followed stringent privacy protection regulations and received approval from the Medical Ethics Committee of Luohe Central Hospital in November 2018 (No. MEC-2018-076). To encourage more pregnant women with positive NIPT/NIPT-plus results to undergo confirmatory invasive testing for accurate diagnosis of fetal chromosomal abnormalities, we provided a commercial insurance policy to each woman who underwent NIPT/NIPT-plus screening. In the event of a positive result from the NIPT/NIPT-plus screening, the insurance company would cover the costs of the subsequent procedures such as amniocentesis, fetal chromosomal karyotyping, or fetal CNV-Seq testing. If an FN result was detected in the NIPT/NIPT-plus screening after birth, the insurance company would provide compensation of 400,000 RMB. This would help encourage participants to report FN results.

### NIPT/NIPT-plus

A total of 10 mL of blood was drawn from each subject, and the "two-step centrifugation method" was used to separate the plasma [26] within 96 hours after blood collection. The plasma cffDNA extraction kit, NGS library construction kit, and fetal chromosomal aneuploidy testing kit used in NIPT/NIPT-plus were manufactured by Berry Genomics Co., Ltd. in Beijing, China. In accordance with the manufacturer’s instructions, cffDNA extraction, NGS library construction, and NGS library quality control were conducted, and gene libraries were sequenced via a NextSeq CN500 sequencer (Illumina, San Diego, CA, USA). Chromosomal aneuploidy and other chromosomal abnormalities were detected by matching the gene sequences obtained from the sequencer with the human reference genome sequence GRCh37 (hg19). Z scores were used to evaluate aneuploidy in chromosomes as follows: when │Z│≥3, the chromosome was considered at high risk for triploidy, whereas when │Z│<3, the chromosome was considered at low risk for triploidy [22]. CNVs were determined via hidden Markov models (HMMs) [27]. The determination of CNVs pathogenicity relies on the 2019 ACMG-ClinGen Copy Number Variant Pathogenicity Classification Guidelines [28]. The laboratory will only inform pregnant women of CNVs rated as " pathogenic" or " likely pathogenic" and recommend follow-up confirmatory invasive testing. The main data quality control standards included the following: the percentage of cffDNA in maternal plasma (cffDNA%) ≥ 4%; the proportion of clean sample data reaching Q30 (Clean Q30) ≥ 80%; the number of unique mapped sequences (Uniq Reads) for NIPT ≥ 1.5 million per sample; and the Uniq Reads for NIPT-plus ≥ 10 million per sample.

### Confirmatory invasive testing

All pregnant women with positive NIPT/NIPT-plus results were advised to undergo confirmatory invasive testing to confirm whether the fetus had any chromosomal abnormalities. Pregnant women who had positive results for common trisomies, SCAs, and RAAs were advised to undergo amniocentesis and fetal chromosomal karyotyping analysis. Similarly, those who tested positive for CNVs were advised to consider amniocentesis and amniotic fluid CNV-Seq testing.

#### Amniocentesis

After 18 weeks of pregnancy, pregnant women were subjected to a procedure in which 20 mL of amniotic fluid was extracted for laboratory diagnosis. Quantitative fluorescence polymerase chain reaction (QF‒PCR) was used for the rapid diagnosis of common chromosomal aneuploidies in all prenatally diagnosed pregnant women, with the exclusion of maternal histocyte or blood contamination in the amniotic fluid. Karyotyping or CNV-Seq results from amniotic fluid are considered the gold standard for fetal chromosomal diagnosis.

#### Chromosome karyotype analysis

A total of 10 mL of amniotic fluid was inoculated into amniotic fluid culture medium (DaHui Biotech, Guangzhou, China; BI, Beit Haemek, Israel), followed by cultivation, harvesting, staining, and karyotype analysis. The karyotype was scanned and analyzed via a fully automated chromosome karyotype analysis system (Carl Zeiss, Jena, Germany). The chromosomal karyotype was described in accordance with the International System for Human Cytogenomic Nomenclature 2019 (ISCN 2019). For each sample, at least 5 different karyotypes were analyzed, and at least 20 karyotypes were counted.

#### CNV-seq

The kits for detecting CNVs, NGS library construction, and DNA purification were all produced by Berry Genomics Co., Ltd., in Beijing, China. In accordance with the manufacturer’s instructions, cffDNA extraction, NGS library construction, and NGS library quality control were conducted, and NGS libraries were sequenced via a NextSeq CN500 sequencer (Illumina, San Diego, CA, USA). The sequencing data were analyzed using the Xromate® analysis system (Berry Genomics), with GRCh37 (hg19) being utilized as the human reference genome sequence. The Xromate® analysis system (Berry Genomics) was used to analyze the sequencing data, and GRCh37 (hg19) was used as the human reference genome sequence. Public databases, including DGV, DECIPHER, OMIM, ClinGen, and PubMed, were used to determine the pathogenicity of CNVs.

### Clinical follow‑up assessment

Follow-ups were conducted for all pregnant women who had undergone NIPT/NIPT-plus, and postpartum follow-ups were carried out via telephone three months after the expected delivery date. The follow-ups included information on the delivery, newborn outcomes, and the physical examination results of the newborn. It was suggested that pregnant women with low-risk NIPT/NIPT-plus results and negative confirmatory invasive testing undergo routine prenatal care and regular ultrasound examinations. Pregnant women with positive results from confirmatory invasive testing were allowed to choose whether to undergo TOP or continue with the pregnancy.

### Statistical analysis

The positive rate (PR), sensitivity, specificity, and PPV of NIPT/NIPT-plus were determined on the basis of the results of NIPT/NIPT-plus and the confirmatory invasive testing to evaluate the screening efficiency. The differences in screening efficiency between NIPT and NIPT-plus were compared. Confirmatory invasive testing results were used as the gold standard for data analysis. The data analysis excluded patients who did not undergo confirmatory invasive testing or were lost to follow-up. All data analyses were conducted via SPSS 25.0 software (SPSS Inc., Chicago, IL, USA). All descriptive data are presented as mean ± SD, while count data are represented as proportions. The chi-square test was used to determine the statistical significance between two categorical variables, with P < 0.05 indicating a significant difference. For the data analysis, PPV = TP/(TP+FP); sensitivity = TP/(TP+FN); and specificity = TN/(TN+FP) (TP: true positive; TN: true negative).

## Results

### General characteristics of the pregnant women undergoing NIPT/NIPT-plus

Table 1 displays the fundamental details of the pregnant women in both the NIPT and NIPT-plus groups. There were no statistically significant differences in the proportion of advanced maternal age (AMA; ≥35 years) (*p* = 0.130) and gestational age (GA) at sampling (*p* = 0.059) between the NIPT and NIPT-plus groups. In the NIPT-plus group, the proportion of pregnant women who have received higher education is higher than that in the NIPT group (47.62% vs. 44.01%; p<0.001), indicating that pregnant women with higher education are more likely to opt for NIPT-plus. The proportion of pregnant women who underwent NIPT-plus at the prenatal diagnosis center was greater than that of those pregnant women whose blood was drawn at the collaborating partner prenatal screening institutions, and the difference was statistically significant (*p*<0.001). Pregnant women with more years of education and those who had received more comprehensive genetic counseling services had a higher acceptance rate of NIPT-plus.

**Table 1:** Demographic and clinical characteristics of the pregnant women undergoing NIPT/NIPT-plus.

Characteristic		NIPT	NIPT-plus
		*n* = 6792	*n* = 5237
*Hospital for blood draw*			
	Partner prenatal screening institution	4231(62.29%)	1849(35.31%)
	Prenatal diagnosis center	2561(37.71%)	3388(64.69%)
*Received higher education*			
	No	3803(55.99%)	2743(52.38%)
	Yes	2989(44.01%)	2494(47.62%)
*GA at sampling (weeks)*			
	≤13	308(4.53%)	232(4.43%)
	14–27	5687(83.73%)	4315(82.39%)
	≥28	797(11.73%)	690(13.18%)
*High-risk group*		3663(53.93%)	3137(59.90%)
	AMA (≥ 35 year)	2118(31.18%)	1691(32.29%)
	AMSS	1337(19.68%)	1234(23.56%)
	UA	161(2.37%)	159(3.04%)
	PFA	47(0.69%)	53(1.01%)
*Low-risk group*		3129(46.07%)	2100(40.10%)
	Routine screening	2610(38.43%)	1708(32.61%)
	Twin pregnancy	225(3.31%)	191(3.65%)
	IVF	294(4.33%)	201(3.84%)

GA, gestational age; AMA, advanced maternal age; AMSS, abnormal maternal serum screening (high risk: T21 ≥ 1/270, T18 ≥ 1/350 and intermediate risk: 1/1000 ≤ T21 ≤ 1/270, 1/1000 ≤ T18 ≤ 1/350); UA, ultrasonic anomalies; PFA, previous fetus/child with abnormalities; IVF, in vitro fertilization.

The pregnant women were categorized into high-risk groups and low-risk groups on the basis of their clinical characteristics recorded during screening. The high-risk group’s clinical characteristics consisted of AMA, abnormal maternal serum screening results (AMSS; high-risk boundary: T21 ≥ 1/270, T18 ≥ 1/350 and intermediate-risk boundary: 1/1000 ≤ T21 ≤ 1/270, 1/1000 ≤ T18 ≤1/350), ultrasonic anomalies (UA), and previous fetuses/children with abnormalities (PFA). On the other hand, the clinical characteristics of the low-risk group included routine screening, twin pregnancies, and in vitro fertilization (IVF). Routine screening was the most common clinical characteristic in both the NIPT and NIPT-plus groups, accounting for 38.43% and 32.61% of the groups, respectively. These findings suggest that NIPT and NIPT-plus have become first-line prenatal screening technologies. AMA (31.18% and 32.29%) and AMSS (19.68% and 23.56%) were the next most prevalent characteristics (Table 1).

### Performance of NIPT/NIPT-plus for screening common trisomies and SCAs

The NIPT group had a PR of 1.37% (93/6792), with 44 (0.65%) positive screenings for common trisomies, 29 (0.43%) positive screenings for SCAs, and 20 (0.29%) positive screenings for RAAs. In contrast, the NIPT-plus group had a PR of 2.10% (110/5237), with 35 (0.67%) positive screenings for common trisomies, 24 (0.46%) positive screenings for SCAs, 16 (0.31%) positive screenings for RAAs, and 35 (0.67%) positive screenings for high-risk CNVs.

The NIPT group identified a total of 44 cases of common high-risk trisomies, including 28 cases of T21, 11 cases of T18, and 5 cases of T13. Additionally, there were 29 cases of high-risk SCAs, including 12 cases of 45,X; 4 cases of 47,XXX; 9 cases of 47,XXY; and 4 cases of 47,XYY. Among the 73 high-risk pregnant women with common trisomies and SCAs in the NIPT group, 71 underwent confirmatory invasive testing. The results revealed that 23 patients were diagnosed with T21, 6 with T18, 1 with T13, and 7 with SCAs. The PPVs for T21, T18, T13, and SCA detection through NIPT screening were 82.14%, 66.67%, 20.00%, and 24.14%, respectively. The NIPT-plus group included 35 cases of high-risk common trisomies, including 23 cases of T21, 8 cases of T18, and 4 cases of T13. Additionally, there were 24 cases of high-risk SCAs, including 9 cases of 45,X; 3 cases of 47,XXX; 9 cases of 47,XXY; and 3 cases of 47,XYY. Among the 59 high-risk pregnant women in the NIPT-plus group with common trisomies and SCAs, 57 underwent confirmatory invasive testing. The results revealed that 20 had T21, 4 had T18, 1 had T13, and 5 had SCAs. The PPVs for screening for T21, T18, T13, and SCAs through NIPT-plus screening were 86.96%, 50.00%, 33.33%, and 21.74%, respectively (refer to Table 2).

**Table 2:** Performance of NIPT/NIPT-plus in screening for fetal chromosome abnormalities.

Chromosomal abnormality		NIPT/NIPT-plus positive		Confirmatory invasive testing		TP	FP	PPV	Sensi- tivity	Speci- ficity
		*n*	%	*n*	%	*n*	*n*	%	%	%
*NIPT*										
*Common trisomies*		44	0.65	42	95.45	30	12	71.43	100	99.82
	T21	28	0.41	28	100.00	23	5	82.14	100	99.93
	T18	11	0.16	9	81.82	6	3	66.67	100	99.96
	T13	5	0.07	5	100.00	1	4	20.00	100	99.94
*SCAs*		29	0.43	29	100.00	7	22	24.14	100	99.68
	45,X	12	0.18	12	100.00	2	10	16.67	100	99.85
	47,XXX	4	0.06	4	100.00	1	3	25.00	100	99.96
	47,XXY	9	0.13	9	100.00	3	6	33.33	100	99.91
	47,XYY	4	0.06	4	100.00	1	3	25.00	100	99.96
*RAAs*		20	0.29	15	75.00	1	14	6.67	100	99.79
*Total*		93	1.37	86	92.47	38	48	44.19	100	99.29
*NIPT-plus*										
*Common trisomies*		35	0.67	34	97.14	25	9	73.53	100	99.83
	T21	23	0.44	23	100.00	20	3	86.96	100	99.94
	T18	8	0.15	8	100.00	4	4	50.00	100	99.92
	T13	4	0.08	3	75.00	1	2	33.33	100	99.96
*SCAs*		24	0.46	23	95.83	5	18	21.74	100	99.66
	45,X	9	0.17	9	100.00	0	9	0.00	NA	99.83
	47,XXX	3	0.06	2	0.6667	1	1	50.00	100	99.98
	47,XXY	9	0.17	9	100.00	3	6	33.33	100	99.89
	47,XYY	3	0.06	3	100.00	1	2	33.33	100	99.96
*RAAs*		16	0.31	13	81.25	0	13	0.00	NA	99.75
*CNVs*		35	0.67	32	91.43	14	18	43.75	100	99.66
*Total*		110	2.10	102	92.73	44	58	43.14	100	98.88

TP, true positive; FP, false positive; PPV, positive predictive value; T21, trisomy 21; T18, trisomy 18; T13, trisomy 13; SCAs, sex chromosome aneuploidies; RAAs, rare autosomal aneuploidies; CNVs, copy number variants; NA, not applicable.

The PPVs for the four SCAs detected by NIPT and NIPT-plus were respectively as follows: 47,XXX (25.00% and 50.00%); 47,XXY (33.33% and 33.33%); and 47,XYY (25.00% and 33.33%), with higher PPVs than for 45,X (16.67% and 0.00%) (Table 2).

NIPT and NIPT-plus showed similar composite PRs (0.65% vs. 0.67%, *p* = 0.890) and composite PPVs (71.43% vs. 73.53%; *p* = 0.839) in screening for common trisomies. Similarly, they also demonstrated comparable composite PRs (0.43% vs. 0.46%; *p* = 0.798) and composite PPVs (24.14% vs. 21.74%; *p* = 0.838) in screening for SCAs. Both NIPT and NIPT-plus screening had a sensitivity of 100% and a specificity of over 0.99 for common trisomies and SCAs. For both common trisomies and SCAs, both NIPT and NIPT-plus had no detected cases of false-negatives. These findings demonstrate that both NIPT and NIPT-plus are effective in screening for common trisomies and SCAs. Despite NIPT-plus having at least 5 times more sequencing data than NIPT does, both tests show similar effectiveness in screening for common trisomies and SCAs.

### Performance of NIPT/NIPT-plus for screening RAAs and CNVs

Among the 6792 cases of NIPT, 20 cases were identified as high risk for RAAs (0.29%, 20/6792), with 15 cases undergoing confirmatory invasive testing. Among them, one patient was diagnosed with mosaic trisomy 20 (T20) (47,XX,+20[44]/,46,XX[54]), resulting in a PPV of 6.67% (Table 2). In a total of 5237 NIPT-plus cases, 16 cases were identified as high-risk RAAs (0.31%, 16/5237). Among them, 13 patients underwent confirmatory invasive testing, all of which were FPs, with a PPV of 0.00%. NIPT-plus identified high-risk CNVs in 35 patients (0.67%, 35/5237), with 32 patients undergoing fetal chromosomal karyotype analysis and CNV-Seq. Among them, 14 cases were confirmed to have pathogenic CNVs, resulting in a PPV of 43.75%. In screening for RAAs, the performance of both NIPT-plus and NIPT was lacking, with a composite PPV of just 3.57%, which is much lower than that for common trisomies and CNVs.

### Performance of NIPT/NIPT-plus for screening chromosomal abnormalities in groups at different risk levels

Among the 6792 pregnant women who underwent NIPT screening, 3663 cases (53.93%) were classified into the high-risk group, whereas 3129 cases (46.07%) were classified into the low-risk group. Among the 5237 pregnant women who underwent NIPT-plus screening, 3137 cases (59.90%) were in the high-risk group, and 2100 cases (40.10%) were in the low-risk group. The proportion of high-risk pregnant women who underwent NIPT-plus was significantly greater than that who underwent NIPT (59.90% vs. 53.93%, *p*<0.001) (Table 1).

The performance of NIPT and NIPT-plus in the high-risk and low-risk groups was calculated separately, as shown in Table 3. In both the NIPT-plus and NIPT groups, the PR in the high-risk group was significantly greater than that in the low-risk group (NIPT: 1.64% vs. 1.05%; *p* = 0.039; NIPT-plus: 2.42% vs. 1.62%; *p* = 0.047). In the NIPT group, the PPV of the high-risk group was significantly greater than that of the low-risk group (51.72% vs. 28.57%; *p* = 0.043). However, in the NIPT-plus group, although the PPV of the high-risk group was greater than that of the low-risk group, the difference was not statistically significant (43.47% vs. 42.42%; *p* = 0.920).

**Table 3:** Performance comparison of NIPT/NIPT-plus between the high-risk and low-risk groups.

Performance	NIPT			NIPT-plus		
	High-risk group	Low-risk group	*p* value	High-risk group	Low-risk group	*p* value
Test (*n*)	3663	3129	NA	3137	2100	NA
Screen positive (*n*)	60	33	NA	76	34	NA
Positive rate (%)	1.64	1.05	0.039	2.42	1.61	0.047
Confirmatory invasive testing (*n*)	58	28	NA	69	33	NA
TP (*n*)	30	8	NA	30	14	NA
FP (*n*)	28	20	NA	33	25	NA
PPV (%)	51.72	28.57	0.043	43.47	42.42	0.920

TP, true positive; FP, false positive; PPV, positive predictive value; NA, not applicable.

### Pregnancy outcomes of the NIPT/NIPT-plus true-positive patients and follow-up results

Follow-up was conducted for all pregnant women who underwent NIPT/NIPT-plus, with 29 cases (NIPT: 16; NIPT-plus: 13) of negative screening pregnant women lost to follow-up.

Pregnancy outcomes for the NIPT/NIPT-plus TP patients confirmed through invasive testing are detailed in Table 4. There were 38 confirmed TP instances in the NIPT group, with 30 instances of common trisomies. As a result, all pregnant women opted for TOP. Among the 7 instances of TP SCAs, one pregnant woman with 47,XXY experienced pregnancy loss at 23 weeks. Three pregnant women (with 47,XXY; 45,X; and 47,XYY) chose TOP. Moreover, three other pregnant women (with 47,XXX; 45,X; and 47,XXY) decided to continue their pregnancies and successfully delivered. There was 1 TP instance of RAAs, which was a chimeric T20, and the pregnant woman opted for TOP.

**Table 4:** Pregnancy outcomes of true-positive instances in NIPT/NIPT-plus.

Chromosomal abnormality		NIPT					NIPT-plus				
		TP	Outcome of TP instances				TP	Outcome of TP instances			
			PL	TOP	LB	LF		PL	TOP	LB	LF
*Common trisomies*		30	0	30	0	0	25	0	25	0	0
	T21	23	0	23	0	0	20	0	20	0	0
	T18	6	0	6	0	0	4	0	4	0	0
	T13	1	0	1	0	0	1	0	1	0	0
*SCAs*		7	1	3	3	0	5	0	3	2	0
	45, X	2	0	1	1	0	0	0	0	0	0
	47, XXX	1	0	0	1	0	1	0	0	1	0
	47, XXY	3	1	1	1	0	3	0	2	1	0
	47, XYY	1	0	1	0	0	1	0	1	0	0
*RAAs*		1	0	1	0	0	0	0	0	0	0
*CNVs*		0	0	0	0	0	14	1	12	1	0
*Total*		38	1	34	3	0	44	1	40	3	0

TP, true positive; PL, pregnancy loss; TOP, termination of pregnancy; LB, live birth; LF, loss follow-up; T21, trisomy 21; T18, trisomy 18; T13, trisomy 13; SCAs, sex chromosome aneuploidies; RAAs, rare autosomal aneuploidies; CNVs, copy number variants.

Among the 44 confirmed TP cases in the NIPT-plus group, 25 were common trisomies, and all pregnant women chose TOP. Among the 5 TP cases of SCAs, 3 chose TOP (2 cases of 47,XXY and 1 case of 47,XYY), whereas 2 chose to continue the pregnancy and successfully gave birth (1 case of 47,XXX and 1 case of 47,XXY). There were no TP cases of RAAs. Among the 14 TP cases of CNVs, one case of 15q13.3 deletion syndrome resulted in pregnancy loss at 19 weeks of gestation. Another patient with 22q11 deletion syndrome continued the pregnancy and gave birth, while the remaining 12 patients chose TOP. Table 5 summarizes the 14 TP CNV cases detected by NIPT-plus and CNV-Seq, with 22q11 deletion syndrome being the most common.

**Table 5:** Fourteen instances of true-positive CNVs detected by NIPT-plus.

Instance	MA (years)	NIPT-plus result	CNV-Seq	Syndrome	Pregnancy outcome
1	31	Dup (1) (q43-q44); size: 3.6 M	Dup (1) (q43-q44); size: 3.36 M	NA	TOP
2	29	Dup (2) (q37.3); size: 2.1 M	Dup (2) (q37.3); size: 2.2 M	NA	TOP
3	36	Dup (6) (q12); size: 3.4 M	Dup (6) (q12); size: 3.44 M	NA	TOP
4	26	Dup (22) (q11.21); size: 2.4 M	Dup (15) (q13.3); size: 0.5 M	NA	Live birth
5	34	Del (15) (q13.3-q14); size: 2.9 M	Del (15) (q13.3-q14); size: 3.0 M	15q13.3 deletion syndrome	Pregnancy loss
6	25	Dup (1) (q31.3-q32.1); size: 4.9 M	Dup (1) (q31.3-q32.1); size: 4.84 M	NA	TOP
7	29	Dup (1) (q43); size: 2.5 M	Dup (1) (q43); size: 2.46 M	NA	TOP
8	30	Del (6) (q24.1); size: 2.2 M	Del (6) (q24.1); size: 1.74 M	NA	TOP
9	33	Dup (4) (q12-q13.1); size: 5.51 M	Dup (4) (q12-q13.1); size: 4.5 M	Hypereosinophi- lic syndrome	TOP
10	27	Del (13) (q33.2-q34); size:8.53 M	Del (13) (q33.1-q34); size: 10.5 M	13q14 deletion syndrome	TOP
11	33	Del (22) (q11.21); size: 2.4 M	Del (22) (q11.21); size: 2.08 M	22q11 deletion syndrome	TOP
12	32	Del (22) (q11.21); size: 3.37 M	Del (22) (q11.21); size: 2.12 M	22q11 deletion syndrome	TOP
13	27	Del (2) (q37.3); size: 3.5 M	Del (2) (q37.3); size: 3.0 M	2q37 monosomy	TOP
14	37	Del (15) (q25.2); size: 3.0 M	Del (15) (q25.2-q25.3); size: 2.97 M	NA	TOP

MA, maternal age; CNV-Seq, copy number variation sequencing; Del, deletion; Dup, duplication; TOP, termination of pregnancy; NA, not applicable.

Seven pregnant women, 2 instances of T18, 1 instance of trisomy 2 (T2), 2 instances of trisomy 8 (T8), 1 instance of trisomy 9 (T9), and 1 instance of trisomy 16 (T16), had positive NIPT results but declined confirmatory invasive testing (Table 6). Two instances of T18 opted for TOP due to fetal ultrasound abnormalities. Subsequent QF‒PCR analysis of fetal skin tissue confirmed the TP for T18. The other 5 patients continued their pregnancies and delivered healthy babies, with no abnormalities detected during prenatal ultrasound or postnatal follow-up. Eight NIPT-plus-positive pregnant women did not undergo confirmatory invasive testing, including 1 instance of T13, 1 instance of 47,XXX, 1 instance of T8, 1 instance of T9, 1 instance of trisomy 22 (T22), 1 instance of CNV-Dup (9)(q12-q21.11), 1 instance of CNV-Del (7)(q1.12-q2.3), and 1 instance of CNV-Dup (4)(q12-q13.1). Among them, 1 patient with T13 showed fetal ultrasound structural abnormalities before prenatal diagnosis, resulting in pregnancy loss. Subsequent QF‒PCR confirmed that the fetus had T13. The remaining instances continued their pregnancies and delivered live births, with no abnormalities detected via prenatal ultrasound and follow-up results.

**Table 6:** Prenatal ultrasound and follow-up outcomes of 15 NIPT/NIPT-plus positive patients who refused prenatal diagnosis.

Instance	NIPT/NIPT-plus result	Prenatal ultrasound	Pregnancy outcome	Fetal outcome
1	NIPT: T18	Choroid plexus cysts; Cystic hygromata; Ventricular septal defect; Hydrops	TOP	*T18
2	NIPT: T18	Omphaloceles; Clenched hands; Hydrops; Strawberry-shaped skull	TOP	*T18
3	NIPT-plus: T13	Ventricular septal defect; Strong echogenic plaque in the bilateral eyeballs; Hyperamniotic fluid	Pregnancy loss	*T13
4	NIPT-plus: 47,XXX	Normal	Live birth	Normal
5	NIPT: T2	Normal	Live birth	Normal
6	NIPT: T8	Normal	Live birth	Normal
7	NIPT: T8	Strong echoes around the ventricles	Live birth	Normal
8	NIPT-plus: T8	Normal	Live birth	Normal
9	NIPT-plus: T9	Normal	Live birth	Normal
10	NIPT: T9	Normal	Live birth	Normal
11	NIPT: T16	Normal	Live birth	Normal
12	NIPT-plus: T22	Normal	Live birth	Normal
13	NIPT-plus: Dup (9)(q12-q21.11); size: 6.5 M	Lateral ventricle widening	Live birth	Normal
14	NIPT-plus: Del (7)(q1.12-q2.3); size: 5.8 M	Normal	Live birth	Normal
15	NIPT-plus: Dup (4)(q12-q13.1); size: 5.5 M	Normal	Live birth	Normal

T18, trisomy 18; T13, trisomy 13; T2, trisomy 2; T8, trisomy 8; T9, trisomy 9; T16, trisomy 16; T22, trisomy 22; Del, deletion; Dup, duplication; TOP, termination of pregnancy.

*Diagnosed by quantitative fluorescence polymerase chain reaction.

Among the 11,826 pregnant women identified as low risk in the NIPT/NIPT-plus screening, the follow-up success rate was 99.75% (11,797/11,826). Among the 11,797 women who were not lost to follow-up (NIPT: 6,683; NIPT-plus: 5,114), 4 women (NIPT: 1; NIPT-plus: 3) chose TOP due to ultrasound abnormalities. One pregnant woman (NIPT-plus) chose TOP for reasons unrelated to the baby’s health, whereas two pregnant women (NIPT) experienced pregnancy loss for unknown reasons. The remaining 11,790 instances (NIPT: 6,680; NIPT-plus: 5,110) resulted in live births, and no chromosomal aneuploidy, CNV-related pregnancy ultrasound abnormalities or newborn developmental abnormalities were detected within 3 months of birth.

**Fig 2:**
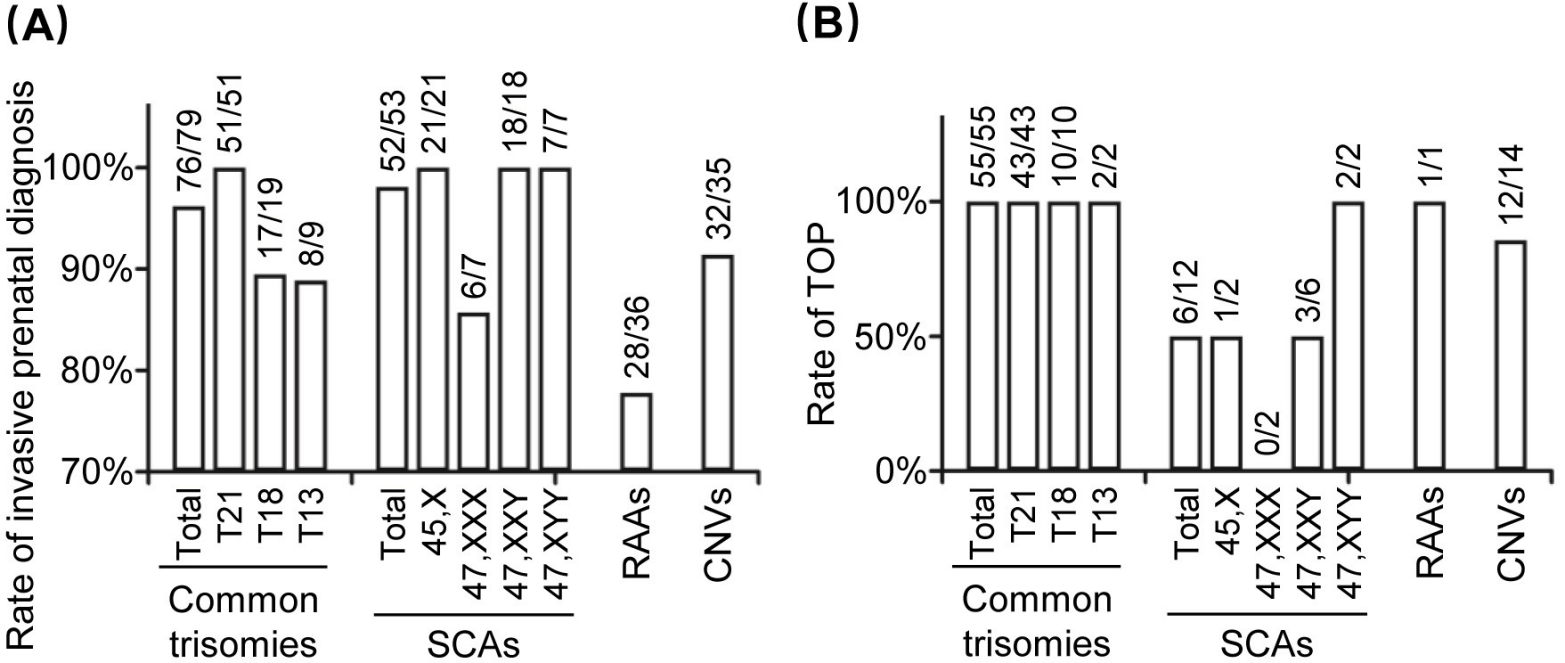
Comparison of the prenatal diagnosis willingness and pregnancy outcomes. a Rate of invasive prenatal diagnosis for women with different NIPT/NIPT-plus positive results. b Rate of TOP for women with different prenatal diagnosis results. T21, trisomy 21; T18, trisomy 18; T13, trisomy 13; SCAs, sex chromosome aneuploidies; RAAs, rare autosomal aneuploidies; CNVs, copy number variants; TOP, termination of pregnancy.

Pregnant women have varying levels of willingness for confirmatory invasive testing for different chromosomal abnormalities, as well as different rates of TOP. A summary of the NIPT and NIPT-plus data shows that pregnant women have higher rates of confirmatory invasive testing for common trisomies, SCAs, and CNVs than they do for RAAs, at 96.20% (76/79), 98.11% (52/53), 91.43% (32/35), and 77.78% (28/36), respectively. Among the TP instances, pregnant women with common trisomies, RAAs, and CNV abnormalities had higher rates of TOP than did those with SCAs, at 100.00% (56/56), 100.00% (1/1), 85.71% (12/14), and 50.00% (6/12), respectively (Fig 2).

## Discussion

Previous studies have shown that 6.0% of fetuses with ultrasound structural abnormalities have clinically relevant CNVs [29]. The development of NIPT-plus has made it possible to screen for pathogenic CNVs non-invasively, although the additional cost of testing is a concern for certain laboratories. Within this research, the quality control standard for sequencing raw data was set at a minimum of 1.5 million unique reads per instance for NIPT and a minimum of 10 million unique reads per instance for NIPT-plus. This means that the sequencing depth or sequencing data cost for NIPT-plus is 6.67 times greater than that of NIPT. In this study, the average number of unique reads for 6792 instances of NIPT was 2.46 million per instance, whereas for 5237 instances of NIPT-plus, it was 12.94 million per instance, which was 5.26 times greater than that of NIPT. When considering NIPT-plus, it is essential to evaluate whether the added cost justifies the enhanced ability to detect chromosomal abnormalities beyond CNVs. By utilizing clinical data, this research evaluated the detection performance of NIPT-plus and NIPT, as well as compared their abilities in detecting common trisomies, SCAs, and RAAs.

The results of this study demonstrate that both NIPT-plus and NIPT have a sensitivity or specificity exceeding 99% for common trisomies, which is consistent with previous research [30, 31]. The PPVs of NIPT or NIPT-plus for T21, T18, and T13 in the literature range from 71%-100%, 48%-85%, and 11%-54%, respectively [23, 32–34]. Our study revealed that the PPVs of NIPT for T21, T18, and T13 were 82.14%, 66.67%, and 20.00%, respectively, whereas the PPVs of NIPT-plus for T21, T18, and T13 were 86.96%, 50.00%, and 33.33%, respectively, which is consistent with the literature. Previous studies have shown a wide range of differences in the reported PPVs of SCAs, ranging from 38.46% to 68.00% [23, 31, 35, 36]. The differences in PPV may be attributed to variations in the methods used to compute biological information from the sequencing results [31]. In our research, the composite PPV of NIPT for SCAs was found to be only 24.14%. The PPVs for the four types of SCAs (45,X; 47,XXX; 47,XXY; and 47,XYY) were 16.67%, 25.00%, 33.33%, and 25.00%, respectively. The composite PPV of NIPT-plus for SCAs was only 21.74%, with individual PPVs for the four types of SCAs being 0.00% for 45,X; 50.00% for 47,XXX; 33.33% for 47,XXY; and 33.33% for 47,XYY. The lower PPV of SCAs in this study might be the result of the small sample size, which could cause bias.

Both NIPT and NIPT-plus have higher PPVs for 47,XXX; 47,XXY; and 47,XYY than for 45,X, which is consistent with previous literature reports [37, 38]. Two main reasons account for the discrepancies in PPVs across different types of SCAs. First, a total of 58 homologous genes are found on both the X chromosome and the Y chromosome, with 29 of these genes located at the end of the sex chromosome. Owing to the short length of cffDNA sequencing, which is only 36 bp, the homologous genes on the X and Y chromosomes are susceptible to sequencing errors [39]. Second, factors such as placental mosaicism, one of the twins being an X monosomy and stopping growth, and maternal X monosomy mosaicism contribute to the differences [37, 40].

Owing to the low incidence rate of RAAs and their tendency toward natural miscarriage in early pregnancy, screening for RAAs via cffDNA is commonly ignored. In this study, the composite PPV of RAAs in the NIPT/NIPT-plus screening was only 3.57%, with other reports also showing that the PPV of RAAs is significantly lower than that of other chromosomal abnormalities[41]. Placental restriction fusion may be a major reason for the high FPR of RAAs [42], and this may be associated with maternal–fetal abnormalities such as preterm birth, fetal growth restriction, and stillbirth [43]. Therefore, pregnant women with positive screening results for RAAs should undergo regular fetal ultrasound monitoring, regardless of whether prenatal diagnosis is performed.

The significant advantage of NIPT-plus lies in its ability to screen for CNVs. In this study, out of 32 NIPT-plus CNV high-risk instances, 14 were confirmed as TP cases, resulting in a sensitivity of 100%, a specificity of 99.73%, and a PPV of 43.75%. In all CNV true-positive cases, instance 4 showed a high-risk result of Dup (22) (q11.21) on NIPT-plus, while confirmatory invasive testing revealed Dup (15) (q13.3).The PPV of NIPT-plus CNVs is greater than that of SCAs and RAAs but lower than that of common trisomies. In this study, the PPV of CNVs was similar to that reported in previous studies [14, 29]; 22q11 deletion syndrome, also known as DiGeorge syndrome, was the most prevalent form of CNV, which is consistent with previous reports [23]. The 3 instances of high-risk 22q11 deletion syndrome discovered in this study were all confirmed as TP cases through prenatal diagnosis, which is consistent with reports of the high PPV for 22q11 deletion syndrome [29]. The screening of CNVs with NIPT-plus can help supplement the lack of resolution in amniotic fluid chromosomal karyotyping, especially for detecting CNVs of 5–10 M [8].

The American College of Obstetricians and Gynecologists (ACOG) recommends the use of NIPT to screen for aneuploidy in pregnant women, regardless of their risk factors (ACOG Committee on Practice Bulletins, 2020). Thus, it is important to evaluate the screening effectiveness of NIPT/NIPT-plus in diverse risk populations of expectant mothers. According to previous reports, NIPT has a greater PPV for screening common aneuploidies or SCAs in the high-risk group than in the low-risk group [36], possibly because of the lower prevalence in the low-risk group. In this study, the PPV of NIPT for the high-risk group was notably greater than that for the low-risk group (51.72% vs. 28.57%, *p* = 0.043). However, for NIPT-plus, although the PPV of the high-risk group was greater than that of the low-risk group, the difference was not statistically significant (43.47% vs. 42.42%, *p* = 0.920) because the calculation of the PPV in the NIPT-plus group included the detection results of CNVs. This calls for our attention, as the range of NIPT screening extends from aneuploidy to CNVs. The traditional "NIPT high-risk group" is changing. The occurrence of pathogenic CNVs is not correlated with maternal age, and neither family history nor maternal age can accurately predict NIPT risk [44]. Consequently, with the expansion of the scope of NIPT screening, the target population for NIPT is also widening.

We also analyzed the willingness of NIPT-positive pregnant women with various types of anomalies to undergo confirmatory invasive testing and the likelihood of TOP, which is affected by factors such as the type of anomaly, level of genetic counseling, cultural practices, etc. [45]. In China, both the confirmatory invasive testing rate and the rate of TOP in women with TP results for common trisomies are significantly high. In this research, a total of 79 instances of high-risk common trisomies were discovered. With the exception of two instances of T18, where noticeable ultrasound abnormalities were detected prior to amniocentesis, leading to TOP, and one instance of T13, resulting in pregnancy loss before amniocentesis, the remaining instances underwent confirmatory invasive testing. Moreover, all confirmed positive instances from invasive testing chose TOP, mirroring the results of Zhou’s study [45]. The confirmatory invasive testing rate for high-risk SCAs reached 98.11% (52/53). Out of the 12 confirmed TP SCA instances, one instance of 47,XXY resulted in a pregnancy loss, whereas six instances (50.00%) opted for TOP. This is a lower proportion than the 61.1% to 81% reported in other studies [23, 45, 46]. Continued pregnancies with TP SCAs consisted of 1 instance of 45,X, along with 2 instances of 47,XXX and 2 instances of 47,XXY, indicating that some pregnant women and their families may accept children with SCAs. Although some individuals with SCAs may have developmental or reproductive issues, their intelligence is generally normal [47]. The confirmatory invasive testing rate for high-risk pregnant women with RAAs was only 77.78% (28/36) because of the high FPR of RAAs and the tendency for early miscarriages to occur with most RAAs. The confirmatory invasive testing rate for high-risk pregnant women with CNVs reached 91.43% (32/35) because all CNVs reported in the NIPT-plus cohort were pathogenic CNVs.

While there are variations in educational background, distribution of blood collection facilities, and percentage of high-risk pregnant women in the NIPT group and NIPT-plus group, comparing the testing performance of NIPT and NIPT-plus in this situation is not rigorous. However, a comparison of the main performance indicators of NIPT and NIPT-plus helps us judge whether NIPT-plus, with increased sequencing depth and cost, has improved detection performance for different types of abnormalities, such as common trisomies, SCAs, and RAAs. The limitations of this study include the following: (1) It is premature to determine whether a newborn has SCAs or CNVs on the basis of follow-up results three months postpartum. Unlike common trisomies, which show obvious signs of chromosomal disorders at birth, symptoms of pathogenic CNVs and SCAs may not appear until childhood [48]. (2) This study excluded screening-positive instances without confirmatory invasive testing results and those lost to follow-up. This exclusion may have an impact on the accuracy of the results and could ultimately affect the conclusions drawn from this study. (3) The samples for this study were collected from eight hospitals in the same region, including a prenatal diagnosis center and seven prenatal screening institutions. The varying levels of professional knowledge and genetic counseling abilities among doctors at different institutions may have an uncertain impact on pregnant women’s choices between NIPT-plus and NIPT, the proportion of prenatal diagnoses, and the management of confirmed fetal abnormalities. (4) Due to limitations of the analysis software, only "pathogenic" or "likely pathogenic" CNVs will be reported, this study did not take into account other types of CNVs, so the overall assessment of CNV detection efficiency is not comprehensive.

## Conclusions

In different groups of pregnant women, both NIPT and NIPT-plus can effectively screen for common trisomies, SCAs, and RAAs. By increasing the amount of sequencing data, NIPT-plus can effectively screen for pathogenic CNVs, but NIPT-plus does not improve the detection performance for common trisomies, SCAs, and RAAs compared with NIPT.

## Supporting information

S1 Dataset (XLS)
